# The Interscalene Triangle: A Cadaveric Dissection of a Critical Neurovascular Corridor at the Root of the Neck

**DOI:** 10.7759/cureus.115760

**Published:** 2026-09-04

**Authors:** Rhyan Ouaddane Alami, Abdesslam Bouassria, Soufiane Mellas, Mustapha Elkouache, Khalid Chakour

**Affiliations:** 1 Laboratory of Anatomy and Surgery, Faculty of Medicine, Pharmacy, and Dental Medicine, Sidi Mohamed Ben Abdellah University, Fez, MAR; 2 Department of Urology, Centre Hospitalier Universitaire Hassan II, Fez, MAR

**Keywords:** brachial plexus, cadaveric dissection, interscalene triangle, phrenic nerve, regional anesthesia, thoracic outlet syndrome

## Abstract

The interscalene triangle is a key cervicothoracic gateway bounded by the anterior and middle scalene muscles and the first rib. It transmits the roots and trunks of the brachial plexus (C5-T1) and the subclavian artery (SA), while the subclavian vein (SV) courses anterior to the anterior scalene muscle and the phrenic nerve (PN) descends along its anterior surface. Cadaveric dissection highlights these critical topographic relationships, providing an anatomical basis for understanding thoracic outlet syndrome, optimizing ultrasound-guided regional anesthesia, and minimizing the risk of iatrogenic nerve injury during cervicothoracic surgery.

## Introduction

The cervicothoracic junction encompasses a complex arrangement of muscular and neurovascular structures that form important anatomical connections between the neck and the upper limb. Among these is the interscalene triangle, a key osteomuscular space bounded by the anterior and middle scalene muscles and by the superior surface of the first rib. It allows the nerve roots from C5 to T1 to pass through and converge toward the primary trunks of the brachial plexus as well as the subclavian artery (SA), whilst the subclavian vein (SV) and the phrenic nerve (PN) lie outside the boundaries of the interscalene space proper [1,2].

A precise understanding of these three-dimensional anatomical relationships is essential for regional anesthetists performing brachial plexus blocks, vascular and thoracic surgeons treating compression of the thoracic outlet, and head and neck surgeons operating in the supraclavicular region. Anatomical variations affecting the scalene muscles, the first rib, and the neurovascular structures may further alter the dimensions and relationships within this region and contribute to neurovascular compression. Cadaveric dissection allows direct visualization of these spatial relationships, thereby complementing cross-sectional imaging and ultrasound [3]. This report presents a detailed cadaveric dissection of the interscalene space, demonstrating both its primary neurovascular contents and the fine collateral branching of the SA and thyrocervical trunk in relation to the scalene musculature.

## Technical report

Materials and methods

Anatomical Material

Dissections were performed on the right side of the neck of one formalin-preserved adult human cadaver at the Laboratory of Anatomy, Faculty of Medicine, Pharmacy, and Dental Medicine, Sidi Mohamed Ben Abdellah University, Fez, Morocco. Specimens with previous neck surgery, major local deformity, or significant pathological alteration of the cervical region were excluded. Unilateral dissection was performed. All our anatomical specimens were derived from body donation programs conducted in accordance with institutional ethical guidelines and national legal frameworks governing medical education and research.

Dissection Technique

The cadaver was placed in a supine position with approximately 30° of cervical extension and 45° of contralateral left rotation, reproducing a conventional surgical position. Standard anatomical instruments were used, including scalpel blades, Metzenbaum and iris scissors, toothed and non-toothed forceps, groove directors, and Farabeuf retractors. The subclavian region was dissected systematically from superficial to deep structures based on classical anatomical methodology.

Results

Cadaveric Dissection and Anatomical Findings

Osteomuscular boundaries and internal neurovascular contents: The interscalene triangle was identified at the root of the neck following meticulous removal of the skin, platysma, and superficial cervical fascia, with lateral retraction of the sternocleidomastoid muscle. The prevertebral layer of the deep cervical fascia was carefully opened to expose the scalene musculature and the neurovascular structures occupying the interscalene space.

The space was bounded anteriorly by the anterior scalene muscle (musculus scalenus anterior); posteriorly by the middle scalene muscle (musculus scalenus medius); and inferiorly by the superior surface of the first rib (costa prima (R1)), particularly the portion posterior to the scalene tubercle.

Within this triangle, the nerve roots from C5 to T1 converged to form the upper (C5-C6), middle (C7), and lower (C8-T1) primary trunks of the brachial plexus. The nerve elements occupied the interscalene sulcus in their characteristic arrangement from top to bottom. Further down, the second part of the SA extended posterior to the anterior scalene muscle, in close relation to the superior surface of the first rib. The spatial arrangement of these structures highlights the close relationship between the nervous and arterial components of the cervicothoracic canal (Figures 1-2).

**Figure 1:**
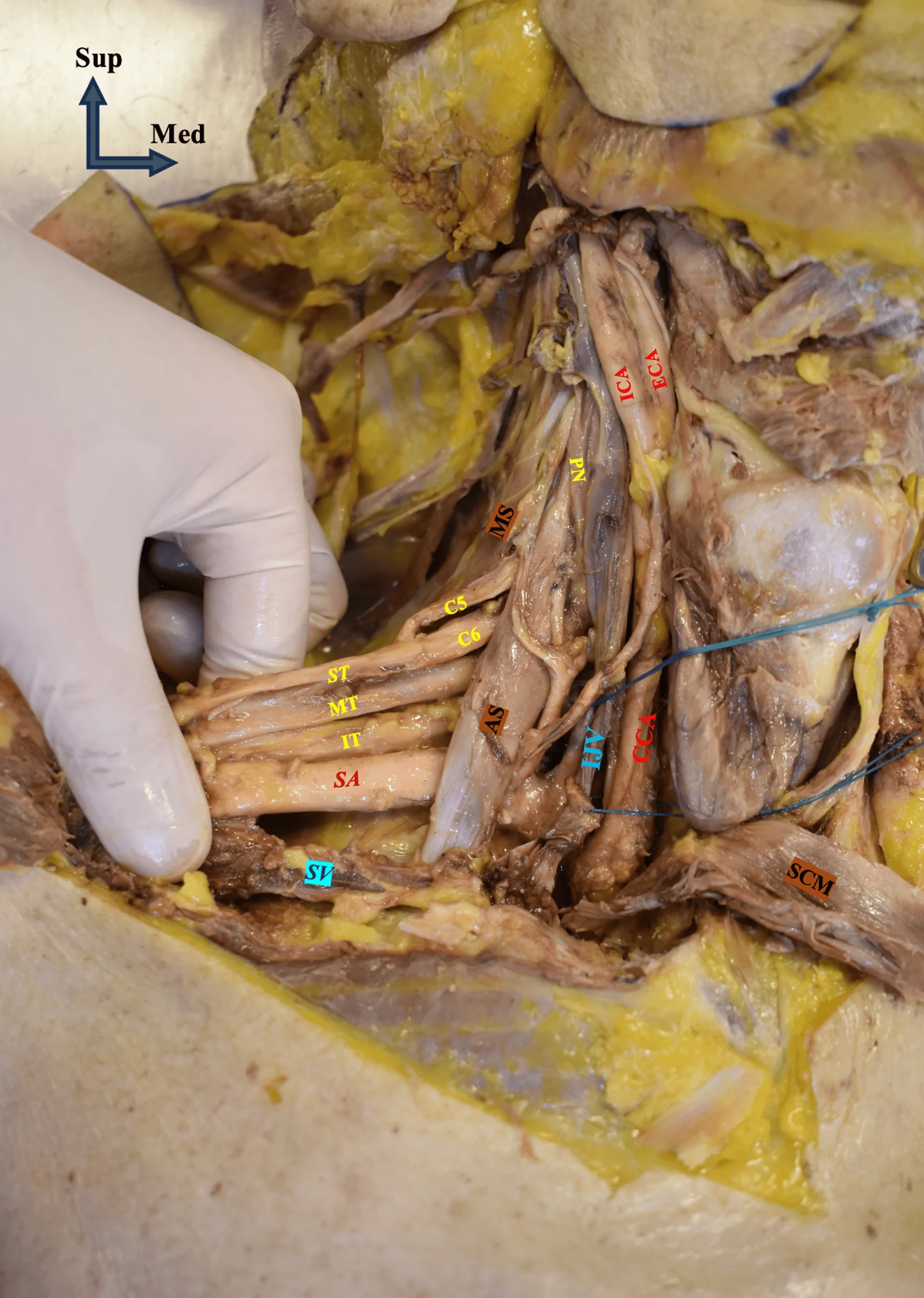
Cadaveric dissection of the interscalene triangle demonstrating its muscular boundaries and neurovascular contents. The anterior scalene (AS) and middle scalene (MS) muscles delimit the triangle, which contains the brachial plexus (BP) trunks - superior trunk (ST), middle trunk (MT), and inferior trunk (IT) - and the subclavian artery (SA). The subclavian vein (SV), located anterior to the AS muscle, and the phrenic nerve (PN), descending along its anterior surface, are shown in their respective relationships. CCA: common carotid artery; ECA: external carotid artery; ICA: internal carotid artery; IJV: internal jugular vein; SCM: sternocleidomastoid muscle; Sup: superior; Med: medial

**Figure 2:**
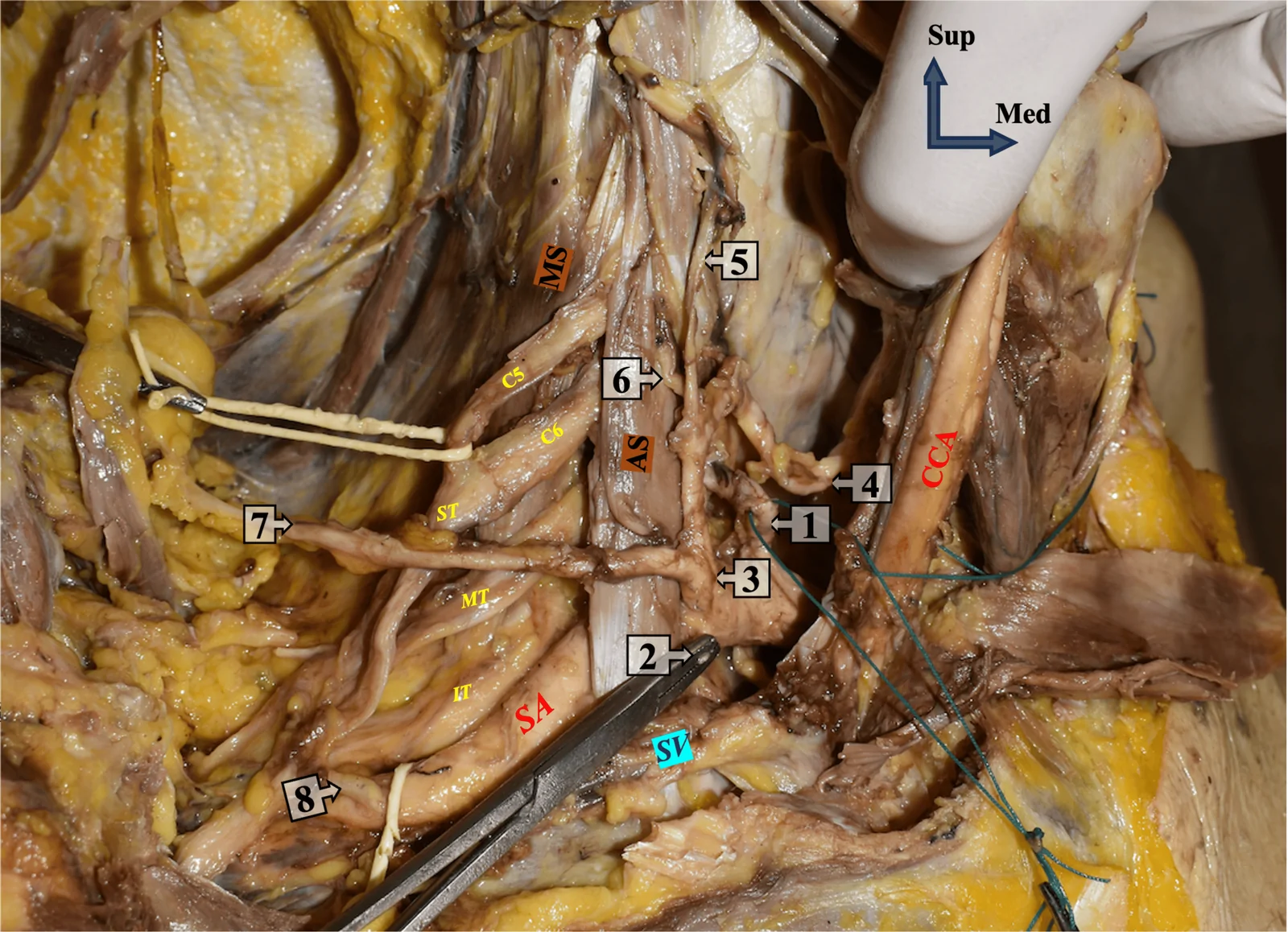
Dissection of the supraclavicular region showing the subclavian artery branches and scalene musculature. The subclavian artery (SA) and its main collateral branches, including the vertebral artery (1), internal thoracic artery (sectioned) (2), thyrocervical trunk (3) giving rise to the inferior thyroid (4), ascending cervical (5), transverse cervical (6), and suprascapular (7) arteries, alongside the dorsal scapular artery (8), are positioned relative to the anterior scalene (AS) and middle scalene (MS) muscles, with the carotid sheath retracted medially. CCA: common carotid artery; SV: subclavian vein; IT: inferior trunk (of the brachial plexus); MT: middle trunk (of the brachial plexus); ST: superior trunk (of the brachial plexus); Sup: superior; Med: medial

Key external topographic relationships: Dissection of the surrounding cervical structures highlighted two clinically important structures lying outside the interscalene triangle proper: the SV and the PN.

The SV was situated in front of the anterior scalene muscle and was therefore located outside the interscalene triangle. The anterior scalene muscle is an important anatomical landmark that separates the SA, situated posteriorly, from the SV, situated anteriorly. This relationship is fundamental to understanding the distinct anatomical pathways of the arterial and venous components of the thoracic outlet [2]. 

The PN was identified arising predominantly from C4, with contributions from C3 and C5, and descending obliquely from lateral to medial across the anterior surface of the anterior scalene muscle. Covered by the prevertebral fascia, the nerve remained outside the interscalene triangle but lay in close anatomical proximity to its anterior boundary. This relationship is of particular importance during procedures involving mobilization or division of the anterior scalene muscle.

Anatomical Variations and Their Clinical Implications

Although no major anatomical variation was identified in the present specimen, several congenital or developmental variations may modify the morphology of the interscalene region and alter its neurovascular relationships [3].

Accessory muscular structures: The presence of a scalenus minimus muscle or accessory fibromuscular bands extending between the lower cervical vertebrae and the first rib may alter the dimensions of the interscalene space and create additional sites of brachial plexus or SA compression [4].

Osseous anomalies: A cervical rib arising from C7 or an elongated C7 transverse process may modify the geometry of the thoracic outlet and contribute to compression of the lower brachial plexus or SA [1,5].

Vascular course variants: Rare anatomical variations may include an atypical course of the SA in relation to the anterior scalene muscle, including partial passage through or anterior to the muscle. Recognition of such variants is particularly relevant during surgical decompression and vascular procedures.

## Discussion

Clinical relevance

Thoracic Outlet Syndrome

The interscalene triangle represents one of the principal anatomical sites where neurovascular compression may occur in thoracic outlet syndrome (TOS), together with the costoclavicular and retropectoralis minor spaces [4,5]. Structural narrowing related to scalene hypertrophy, fibrous bands, cervical ribs, or other osseous abnormalities may compromise the neurovascular structures traversing the region.

Neurogenic TOS (nTOS): Neurogenic TOS is the most common clinical form of TOS and results from compression or irritation of elements of the brachial plexus, often with predominant involvement of the lower plexus. Patients may present with pain and paresthesia along the medial aspect of the upper limb and, in advanced cases, weakness of the intrinsic hand muscles. Severe chronic involvement may result in the characteristic Gilliatt-Sumner hand [6].

Arterial TOS (aTOS): Chronic compression of the SA against the first rib or an anomalous bony structure may result in arterial stenosis, post-stenotic aneurysmal dilatation, mural thrombosis, and distal embolization, potentially leading to ischemic manifestations in the upper limb.

Venous thoracic outlet syndrome, in contrast, generally results from compression of the SV within the costoclavicular compartment rather than within the interscalene triangle [4,5].

Regional Anesthesia

The interscalene region is the anatomical target of the ultrasound-guided interscalene brachial plexus block (ISB), widely used for anesthesia and analgesia during shoulder and proximal upper-limb surgery [6,7].

On transverse ultrasound imaging, the brachial plexus roots and proximal trunks are typically identified between the anterior and middle scalene muscles as a cluster of hypoechoic, rounded neural structures. At appropriate cervical levels, their arrangement may produce the characteristic “stoplight” appearance.

The close anatomical relationship between the brachial plexus and the PN explains one of the principal limitations of conventional interscalene blockade. Because the PN courses on the anterior surface of the anterior scalene muscle, local anesthetic spread may reach the nerve and result in a high incidence of ipsilateral hemidiaphragmatic paresis, particularly with standard-volume techniques [7]. Contemporary phrenic-sparing strategies, including low-volume or extrafascial approaches and targeted superior trunk blocks, rely on precise understanding of these anatomical relationships to reduce respiratory complications.

Surgical Decompression and Neck Surgery

From a surgical perspective, identification and careful preservation of the PN are essential when mobilization or division of the anterior scalene muscle is required. During surgical decompression for thoracic outlet syndrome, including supraclavicular or transaxillary approaches to first-rib resection, meticulous dissection around the interscalene region is necessary to avoid injury to the brachial plexus, subclavian vessels, and PN [8,9].

The relationship between the SA and the first rib is particularly relevant during decompression, while recognition of the anteriorly positioned SV helps define the venous compartment and avoid inadvertent vascular injury. These relationships underscore the importance of a detailed three-dimensional anatomical understanding before surgical manipulation of the scalene muscles or first rib.

Limitations of the study

The findings presented in this report are based on the descriptive, high-resolution cadaveric dissection of a single specimen. As such, this study is inherently limited in its generalizability and does not allow for statistical conclusions regarding the population-wide prevalence of anatomical variations, such as the scalenus minimus muscle, cervical ribs, or vascular anomalies in the supraclavicular region. Further multi-specimen and bilateral quantitative cohort studies are necessary to accurately establish the true frequencies and demographic distributions of these topographical variants.

## Conclusions

The interscalene triangle is a clinically important three-dimensional anatomical corridor through which the brachial plexus and SA pass between the anterior and middle scalene muscles and above the first rib. Cadaveric dissection provides direct visualization of the spatial separation between these structures and the anteriorly positioned SV, while highlighting the close relationship of the PN to the anterior scalene muscle. A precise understanding of these anatomical relationships is essential for the safe performance of regional anesthesia, surgical decompression of thoracic outlet compression, and operative procedures involving the lower neck and cervicothoracic junction.

While these findings provide valuable illustrative insights, they represent a single-specimen observation and are not intended to be generalized across all population-level anatomical variations.
